# CLINICAL TRIALS OF GEROTHERAPEUTICS FOR TARGETING LOWER URINARY TRACT SYMPTOMS

**DOI:** 10.1093/geroni/igae098.2049

**Published:** 2024-12-31

**Authors:** Iman Al-Naggar

**Affiliations:** UConn Health, Farmington, Connecticut, United States

## Abstract

Aging and the metabolic syndrome, a condition that accelerates biological aging, are both risk factors for lower urinary tract symptoms (LUTS). Storage LUTS, including urinary frequency, urgency, incontinence and nocturia, greatly impact quality of life and contribute to social isolation, depression, increased risk of falls and fractures, and loss of independence in older adults. Dysregulated mechanisms along the brain-to-bladder pathway can lead to LUTS, as the body becomes less resilient and its ability to maintain normal function declines. Although dysfunctional detrusor muscle can be targeted with anticholinergics, these drugs have had little success in alleviating LUTS and are often abandoned due to undesirable side effects. Identifying novel targets would allow the development of more effective drugs to alleviate LUTS. Biological mechanisms of aging that play a role in multiple age-related diseases have recently been implicated in metabolic syndrome-associated LUTS, including inflammation, mitochondrial dysfunction and oxidative stress. Furthermore, studies using mouse models of aging and metabolic syndrome show increased bladder dysfunction in these model organisms. Targeting biological hallmarks of aging to alleviate LUTS in humans represents a promising new direction that our group is exploring. This can be safely done using repurposed drugs or supplements with gerotherapeutic properties, such as MitoQ. This symposium session will describe the rationale and study design for on-going pilot and feasibility studies targeting the biology of aging for LUTS alleviation in older women.

